# Probiotics Improve Chemerin Levels and Metabolic Syndrome Parameters in Obese Rats

**DOI:** 10.4274/balkanmedj.galenos.2019.2019.2.61

**Published:** 2019-08-22

**Authors:** Menşure Nur Çelik, Mehtap Ünlü Söğüt

**Affiliations:** 1 Department of Nutrition and Dietetics, Gazi University School of Health Sciences, Ankara, Turkey; 2 Department of Nutrition and Dietetics, Ondokuz Mayıs University School of Health Sciences, Samsun, Turkey

**Keywords:** Chemerine protein, metabolic syndrome, obesity, probiotics, rat

## Abstract

**Background:**

Chemerin is a recently discovered adipokine that plays a role in adipocyte metabolism. It is a novel chemoattractant adipokine whose expression and secretion are increased by adipogenesis.

**Aims:**

To evaluate the effects of probiotic supplementation on chemerin level, inflammation, and metabolic syndrome components in obese Wistar rats.

**Study Design:**

Animal experiment.

**Methods:**

We divided the experimental animals into three groups, each consisting of eight rats. Group 1 was the control group. Group 2 was the experimentally obese group, in which rats were fed with a high-fat diet. Group 3 was the obese intervention group, in which rats were supplemented with probiotics after obesity induction.

**Results:**

At the end of the study, a statistically significant difference was found between the groups in final weights, weight changes, and body mass index values (p<0.05). Weight gain was 34.12±3.70 g in group 3 post-probiotic supplementation and 53.25±8.35 g in group 2 (p<0.05). Obese rats showed increased levels of fasting plasma glucose, insulin, insulin resistance (homeostatic model assessment-insulin resistance), total cholesterol, low-density lipoprotein cholesterol, inflammatory markers, and leptin compared to those in the control group. Chemerin levels were 14.31±13.34 ng/mL in group 2 and 2.67±2.42 ng/mL in group 3 (p<0.05).

**Conclusion:**

Probiotic supplementation (group 3) reduced weight gain, and there were positive effects on the levels of fasting plasma glucose, insulin, homeostatic model assessment-insulin resistance, triglycerides, inflammatory markers, leptin, and chemerin.

The World Health Organization defines obesity as abnormal or excessive fat accumulation caused by an imbalance between energy intake and energy expenditure (1). Worldwide, 390 million adult women and 281 million adult men were reported to be obese in 2016 (2). In recent years, intestinal microflora emerged as important components for the prevention of obesity and related diseases as well as for supporting treatment. Regulation of intestinal microflora (such as by antibiotic, prebiotic, and probiotic use), and the effectiveness of treatment for obesity and related diseases are being investigated using human studies following successful animal studies (3,4,5). Probiotics, which are viable microorganisms with beneficial health effects, when taken in sufficient quantities have the potential to reduce obesity by decreasing fat storage and increasing satiety and energy expenditure (6).

The strong association between diet, intestinal microbiota, and obesity has been an important research topic in recent years to better understand the etiology of obesity and to develop new treatment methods (7). Especially, recent metagenomics-based studies have shown that intestinal microbiota affects not only the energy balance but also the immune and intestinal barrier functions and acts as a factor affecting the entire body metabolism (8,9).

Chemerin is a recently discovered adipokine that has been demonstrated to play a role in adipogenesis and adipocyte metabolism (10,11). Chemerin expression and secretion are known to be increased by adipogenesis (12). Therefore, it is important to determine and regulate circulating levels of chemerin as they are associated with an increased risk of developing obesity, type 2 diabetes mellitus, cardiovascular diseases, inflammation, metabolic syndrome, and several other diseases (13,14).

Studies investigating the relationship between chemerin and obesity have reported that individuals who followed various methods for weight loss have low serum chemerin levels compared to those in obese individuals without weight loss, and the decrease in the serum concentrations of chemerin was found to be associated with the improvement in weight loss and metabolic parameters (15,16,17,18).

Metabolic syndrome is a combination of several cardiovascular risk factors that are closely associated with abdominal adiposity and insulin resistance (18). Circulating chemerin levels are known to increase in individuals with obesity and positively correlate with metabolic syndrome markers, including body mass index, waist/hip ratio, systolic blood pressure, and serum triglyceride levels (19).

In this study, we evaluated the effects of probiotics on diabetes, insulin resistance, lipid profile, obesity, inflammation, and chemerin levels in obese rats.

## MATERIALS AND METHODS

### Experimental animals

The Ondokuz Mayıs University Experimental Animal Application and Research Center supplied 24 male rats aged 4-6 weeks with an average weight of 270-290 g. All rats were housed under standard conditions (such as heat, humidity, light, and ventilation) and fed *ad libitum* with dry chow (20-25 g/day per rat) during the study. All rats were housed in plastic cages at 24 °C±3 °C in 12-h light/12-h dark cycles with 40%-60% humidity in the laboratories of the Ondokuz Mayıs University. Rats had free access to water and food. Ethical approval was obtained from the Ondokuz Mayıs University Ethical Committee of Animal Experiments (IRB number: 28.12.2016/12).

### Experimental design and animal grouping

**Figure 1:**
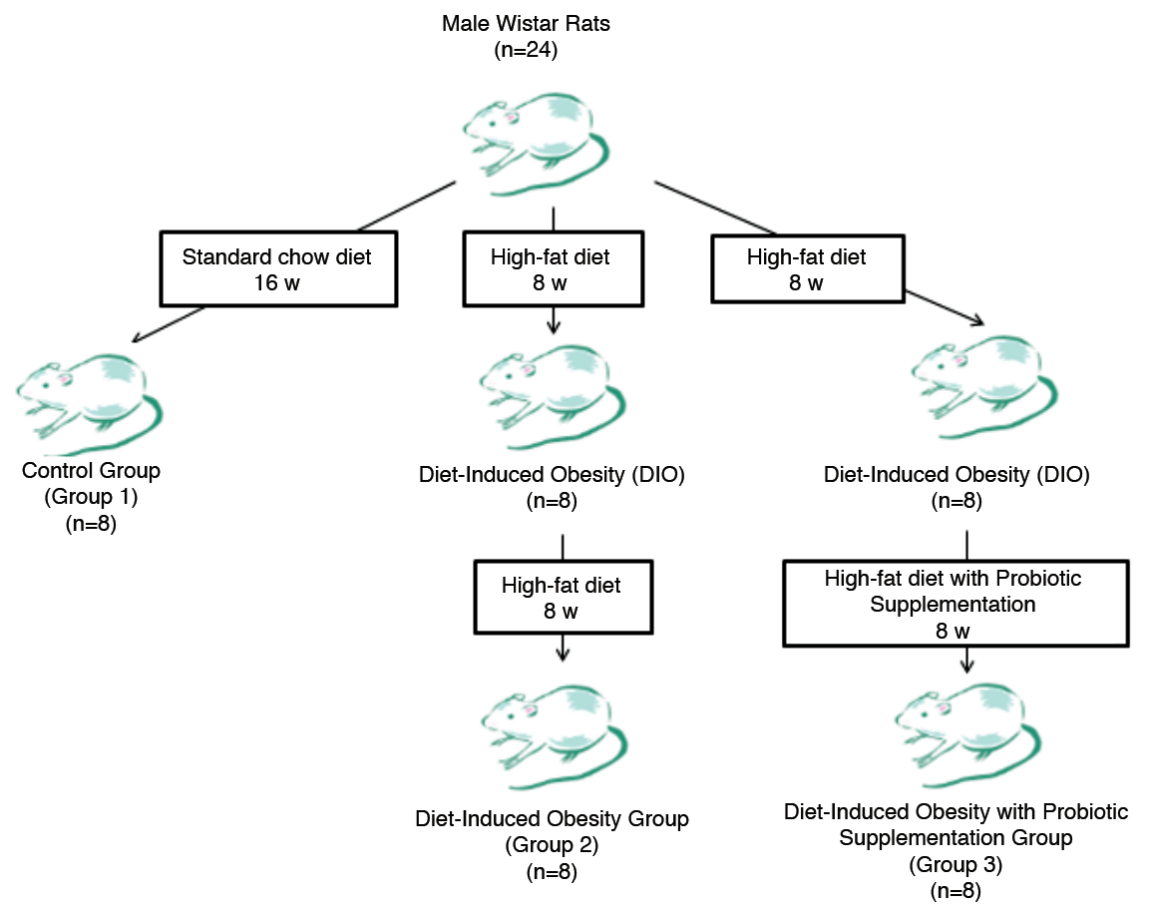
Flow diagram outlining the experimental design.

The experiment lasted 16 weeks (w) and was divided into the following two phases: 1) induction of obesity [diet-induced obesity (DIO)] (0-8 w) and 2) intervention (9-16 w). The 24 rats were randomized into three groups containing eight rats in each group. Group 1 was the control group fed with a standard diet, group 2 was the study group fed with a high-fat diet (HFD) (60% fat), and group 3 was another study group fed with HFD supplemented with probiotics (Figure 1).

During the entire duration of the experiment, body weights of the rats were recorded weekly and mean body weight and weight gain were calculated. For the evaluation of obesity, body mass index [body weight (g)/height (cm^2^)], which was calculated by measuring the body weight and the naso-anal length, was used. Rats with a body mass index >0.68 g/cm^2^ were considered as obese (20).

### DIO rats and diets of animals

Rats in group 1 were fed with standard rat chow (16% carbohydrate, 54% protein, and 30% fat) throughout the study (16 w). Rats in group 2 and group 3 were fed with HFD (8.9% carbohydrate, 30.8% protein, and 60.3% fat) for 8 w to stimulate DIO. After 8 w, DIO rats in group 2 continued eating the HFD. However, the DIO rats in group 3 received probiotic supplementation via oral gavage in addition to the HFD.

### Probiotic administration protocol

A pool of probiotics that included *Lactobacillus acidophilus, Bacillus lactis, Lactobacillus paracasei*, and *Lactobacillus rhamnosus* (Solgar^®^, Advanced Multi-Billion Dophilus™, Turkey) was given for 8 w daily (6×10^8^ of each strain; final concentration 2.4×10^9^ cfu bacteria). Prior to gavage, the probiotics were diluted in sterile water (21).

### Blood samples

The animals were decapitated under anesthesia, and the maximum amount of blood that could be collected directly was used for biochemical analysis. Blood samples were collected in anticoagulant-free biochemical tubes, centrifuged at 3000 rpm for 10 min, and the resulting sera were stored at -80 °C until analysis.

### Biochemical analyses of serum

Insulin and fasting plasma glucose levels were analyzed as an indicator of diabetes; the levels of the cytokines interleukin-6 (IL-6), IL-10, and tumor necrosis factor-α and C-reactive protein were analyzed as indicators of inflammation; leptin level was evaluated as an indicator of obesity; triglyceride, total cholesterol, high-density lipoprotein cholesterol, and low-density lipoprotein cholesterol levels were determined to evaluate the lipid profile; and serum levels of chemerin were analyzed in serum samples. For this analysis, the enzyme-linked immunosorbent assay technique was applied using a commercially available kit (Rel Assay Diagnostics^®^, Turkey).

Insulin resistance was measured using the homeostasis model assessment of insulin resistance= fasting insulin (μU/mL) × fasting glucose (mmol/L)/22.5. An indirect measure of insulin sensitivity was calculated using the quantitative insulin sensitivity check index as follows: 1/[log(fasting insulin μU/mL) + log(fasting glucose mg/dL)].

### Statistical analysis

Data were tested for normal distribution using the Shapiro–Wilk test. When the criteria for normal distribution were not achieved, the nonparametric Kruskal–Wallis and Mann–Whitney U pairwise comparison tests were used. The Mann–Whitney U test was performed to assess the significance of pairwise differences using Bonferroni correction to adjust for multiple comparisons. An overall 5% type-I error level was used to determine the statistical significance. Results are presented as mean ± standard deviations. Values were considered to be significant when p<0.05; different superscript letters (a, b) were used to indicate significant variations at p<0.05 in the tables. Considering leptin values, the minimum number of samples for 95% confidence, a significant difference of 0.55, a standard deviation of 0.35, and a test power of 0.80 was eight rats (per group) (22,23). Statistical analyses were performed using the SPSS (Statistical Package for the Social Sciences) 21 application.

A minimum difference of 0.55, a standard deviation of 0.35, and a test power of 0.80, for 95% confidence, taking into account the minimum number of samples, the leptin value, were eight rats.

## RESULTS

### Evaluation of morphometric measurements

DIO rats were fed with HFD with or without probiotic supplementation for 16 w. Table 1 shows the initial and final weights, body mass index values, and weight changes of the rats.

**Figure 2:**
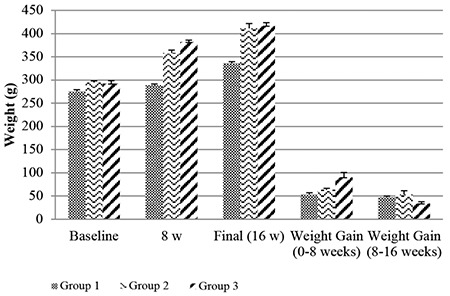
At baseline, there was no difference in body weight between groups. Body weight changes and weight gains at 8 w and 16 w are shown. Group 1: control; Group 2: diet-induced obesity; Group 3: diet-induced obesity with probiotic supplementation

**Figure 3:**
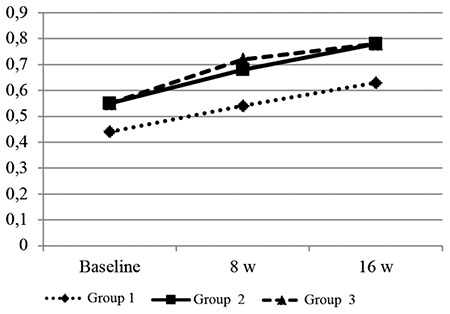
The figure shows that BMI changes between groups. BMI values were rapidly increased in group 2. However, BMI values tended to increase slowly after the start of probiotic supplementation in group 3. Group 1: control; Group 2: diet-induced obesity; Group 3: diet-induced obesity with probiotic supplementation

At baseline, there was no significant difference in body weight between the groups (p>0.05). After the first 8 w, the average weight gain was 63.37±9.69 g in group 2 and 90.50±29.07 g in group 3. Between weeks 8 and 16, the average weight gain was 53.25±23.62 g in group 2 and 34.12±10.46 g in group 3. Weight changes and body mass index values were significantly increased at 8 and 16 w in rats fed with HFD (p<0.05). The changes in weight and weight gain recorded during the study are shown in Figure 2. However, despite having a higher body mass index than the other groups at week 8, group 3 showed a significant decrease in the rate of body mass index increase after receiving probiotics. Figure 3 shows the body mass index changes observed during the study.

### Evaluation of biochemical parameters

To evaluate the effects of HFD and probiotic supplementation on biochemical parameters in DIO rats, we examined the levels of fasting plasma glucose, insulin, inflammatory markers, chemerin, and leptin and the lipid profile. Table 2 shows the biochemical parameters of the rats.

**Figure 4:**
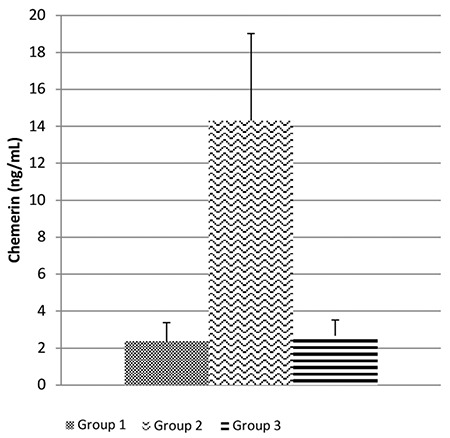
The figure shows that mean levels of chemerin adipokine according to groups. Chemerin significantly increased in response to a HFD (group 2), but significantly recovered after introduction of probiotic supplementation (group 3). Group 1: control; Group 2: diet-induced obesity; Group 3: diet-induced obesity with probiotic supplementation

Insulin levels, insulin resistance measured using homeostasis model assessment of insulin resistance, and insulin sensitivity measured using quantitative insulin sensitivity check index were significantly decreased in rats after probiotic supplementation compared to those in rats fed with HFD without supplementation. Chemerin levels were significantly increased in response to HFD (group 2) but significantly recovered after probiotic supplementation (group 3) (Figure 4). No statistically significant difference was observed in the remaining parameters (p>0.05).

## DISCUSSION

In recent years, it has been suggested that regulation of microbiota by various mechanisms using probiotics plays a role in preventing obesity and related diseases (6).

It has been reported that diets in which 45%-60% of the energy is derived from fats provide 20%-40% of weight gain (24,25). Our results were similar to the literature, and the mean body weight and weight gain (weight increase of 21% at 8 w and 39% at 16 w), due to increased adipogenesis, in DIO rats were higher than those in the control group (p<0.05). Furthermore, rats in group 3 were appeared to have more weight gain than rats in group 2, but the difference was not significant.

Probiotics can affect metabolic syndrome, type 2 diabetes mellitus, and obesity by regulating intestinal microbiota, favoring insulin signaling, and decreasing cholesterol levels. Evidence obtained from animal studies indicates that probiotic supplementation partially reduces weight gain and positively affects parameters related to obesity and HFD-related diseases (26,27). In our study, body weight gain was decreased in DIO rats that received probiotic supplementation (group 3) compared to that in DIO rats (group 2). After probiotic supplementation in our study, the levels of fasting plasma glucose, insulin, and homeostasis model assessment of insulin resistance were found to be consistent with the literature.

HFD causes adverse changes in the lipid profile, which can be normalized after probiotic supplementation (28,29). Some studies observed no effect after probiotic supplementation on total cholesterol, high-density lipoprotein cholesterol, low-density lipoprotein cholesterol, and triglyceride levels (30,31). Results of our study also demonstrated that although there was no significant difference in total cholesterol, low-density lipoprotein cholesterol, and high-density lipoprotein cholesterol levels after probiotic supplementation, the decrease in triglyceride levels was similar to the variable results of the lipid profile reported in the literature.

Research shows that supplementation with probiotics to HFD reduces leptin levels compared with standard diets (32). In a similar study, no difference was observed in leptin levels between groups after 12 weeks of probiotic supplementation (33). In our study, we found that leptin levels were decreased in DIO rats after probiotic supplementation (group 3) compared to those in DIO rats (group 2), although the difference was not significant.

Supplementation of probiotics to rats fed with HFD resulted in an anti-inflammatory response demonstrated by the reduction in the levels of proinflammatory cytokines such as IL-6, IL-17, and tumor necrosis factor-α. Results obtained from animal experiments suggest that supplementation of probiotics to HFD stimulates an anti-inflammatory response (29,32). Another study reported that probiotic supplementation to HFD did not alter tumor necrosis factor-α and IL-6 levels (33). In our study, the levels of IL-6, IL-10, tumor necrosis factor-α, and C-reactive protein were decreased in DIO rats that received probiotic supplementation (group 3) compared to those in DIO rats (group 2), although the difference did not reach statistical significance.

It is known that adipogenesis increases the expression and secretion of the adipokine chemerin (10,12). Studies have shown that rats fed with HFD gained more weight and had higher levels of chemerin than those fed with a standard diet (34,35). Similarly, the weight increase in rats fed with HFD was associated with an increase in chemerin levels in our study. Rats fed with HFD showed higher levels of chemerin than the control group (p<0.05).

Studies have shown that chemerin levels are associated with metabolic syndrome markers (17,36). In our study, increased levels of chemerin were found to be associated with elevated levels of fasting plasma glucose, insulin, homeostasis model assessment of insulin resistance, total cholesterol, high-density lipoprotein cholesterol, and low-density lipoprotein cholesterol and reduced levels of triglyceride in DIO rats. Chemerin levels were decreased in DIO rats after probiotic supplementation (p<0.05).

In conclusion, although this study demonstrates a relationship between chemerin levels and metabolic syndrome components, the effect of probiotic supplementation on serum levels of chemerin, and hence obesity and related diseases, is unknown. No studies have yet investigated the effect of probiotics on chemerin levels, although probiotics are known to be related to diseases and are a current topic under investigation. Therefore, our study contributes to the literature as the first investigation to evaluate these parameters together.

For this purpose, it is important to show that chemerin is a new modifiable factor of obesity affected by probiotic supplementation. Results of this study obtained from obese rats would provide both weight control and reduction risk of developing obesity-related diseases by increasing the levels of chemerin to the desired level with probiotic supplementation in the diet of obese individuals.

## Figures and Tables

**Table 1:**
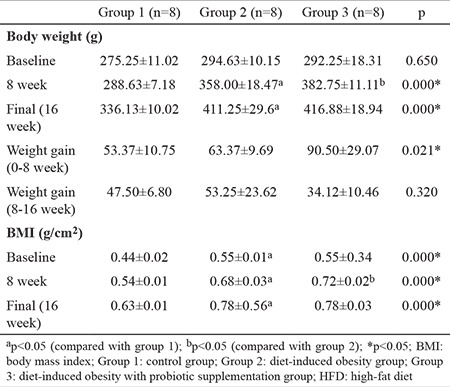
Effects of normal diet and HFD on body weight and BMI in rats

**Table 2:**
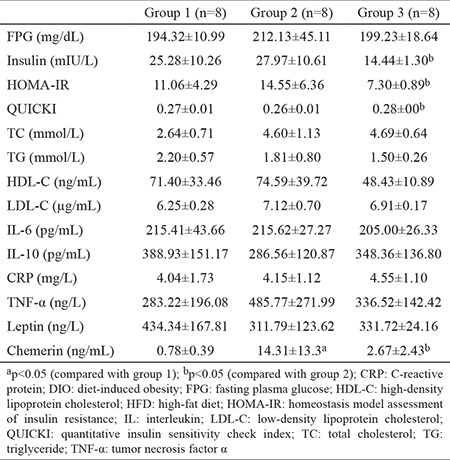
Effects of HFD and probiotic supplementation on biochemical parameters in DIO rats
